# Clinical and Microbiological Features of HIV-Associated Tuberculous Meningitis in Vietnamese Adults

**DOI:** 10.1371/journal.pone.0001772

**Published:** 2008-03-19

**Authors:** M. Estee Torok, Tran Thi Hong Chau, Pham Phuong Mai, Nguyen Duy Phong, Nguyen Thi Dung, Ly Van Chuong, Sue J. Lee, M. Caws, Menno D. de Jong, Tran Tinh Hien, Jeremy J. Farrar

**Affiliations:** 1 Oxford University Clinical Research Unit, Hospital for Tropical Diseases, Ho Chi Minh City, Vietnam; 2 Hospital for Tropical Diseases, Ho Chi Minh City, Vietnam; 3 Mahidol Oxford Tropical Medicine Research Unit, Faculty of Tropical Medicine, Mahidol University, Bangkok, Thailand; 4 University of Oxford, Centre for Clinical Vaccinology and Tropical Medicine, Churchill Hospital, Oxford, United Kingdom; University of Stellenbosch, South Africa

## Abstract

**Methods:**

The aim of this prospective, observational cohort study was to determine the clinical and microbiological features, outcome, and baseline variables predictive of death, in Vietnamese adults with HIV-associated tuberculous meningitis (TBM). 58 patients were admitted to the Hospital for Tropical Diseases in Ho Chi Minh City and underwent routine clinical and laboratory assessments. Treatment was with standard antituberculous therapy and adjunctive dexamethasone; antiretroviral therapy was not routinely available. Patients were followed up until the end of TB treatment or death.

**Results:**

The median symptom duration was 11 days (range 2–90 days), 21.8% had a past history of TB, and 41.4% had severe (grade 3) TBM. The median CD4 count was 32 cells/mm^3^. CSF findings were as follows: median leucocyte count 438×10^9^cells/l (63% neutrophils), 69% smear positive and 87.9% culture positive. TB drug resistance rates were high (13% mono-resistance 32.6% poly-resistance 8.7% multidrug resistance). 17% patients developed further AIDS-defining illnesses. 67.2% died (median time to death 20 days). Three baseline variables were predictive of death by multivariate analysis: increased TBM grade [adjusted hazard ratio (AHR) 1.73, 95% CI 1.08–2.76, p = 0.02], lower serum sodium (AHR 0.93, 95% CI 0.89 to 0.98, p = 0.002) and decreased CSF lymphocyte percentage (AHR 0.98, 95% CI 0.97 to 0.99, p = 0.003).

**Conclusions:**

HIV-associated TBM is devastating disease with a dismal prognosis. CSF findings included CSF neutrophil predominance, high rates of smear and culture positivity, and high rates of antituberculous drug resistance. Three baseline variables were independently associated with death: increased TBM grade; low serum sodium and decreased CSF lymphocyte percentage.

## Introduction

Tuberculous meningitis (TBM) is the most severe form of *Mycobacterium tuberculosis* infection, and carries a high morbidity and mortality [1]. HIV infection is associated with an increased risk of developing tuberculosis (TB), particularly extra-pulmonary disease [2]. The clinical features of TBM mimic other chronic meningoencephalitides, making diagnosis difficult. The classical cerebrospinal fluid (CSF) findings include a lymphocytic CSF pleocytosis, raised protein and low CSF to blood glucose ratio. The diagnosis of TBM usually relies on the detection of acid-fast bacilli in CSF, a simple, affordable method with low diagnostic sensitivity in routine laboratories. Culture of *M. tuberculosis* from the CSF is the diagnostic gold standard but is expensive, requires laboratory infrastructure and expertise, and takes too long to guide initial patient management.

Despite the availability of highly effective antituberculous chemotherapy, the outcome from TBM remains poor [3], [4]. The optimal treatment of TBM has not been determined by randomized controlled trials, although a number of clinical guidelines exist [5]–[7]. Multi-drug resistant tuberculosis (MDR-TB) has long been recognized to be associated with institutional outbreaks in HIV-infected patients [8]. Outside the outbreak setting, the data on the association between HIV infection and MDR-TB is limited [8]. More recently an outbreak of extensively drug resistant tuberculosis (XDR-TB) has been been reported [9]. Patients with MDR-TB have lower cure rates and higher mortality rates that patients with drug susceptible TB [10]–[13].

The treatment of HIV-associated TB is similar to TB in HIV-negative patients with some important differences; these include the drug interactions between rifamycins and antiretrovirals, paradoxical reactions or immune reconstitution inflammatory syndrome (IRIS) [14]–[17] and the potential to develop rifamycin resistance with highly intermittent therapy [18]. Although antiretroviral therapy (ART) is recommended for all HIV patients presenting with TB, the optimal time to initiate ART is unknown [19]. Two retrospective studies have shown that starting ART early in severely immunosuppressed patients with TB is associated with decreased mortality and a lowering of rates of progression [20], [21].

Vietnam ranks 13^th^ out of 22 countries defined as high burden for tuberculosis (TB) by the World Health Organization (WHO), with an estimated incidence of 178/100,000 population per year [22]. The estimated prevalence of multi-drug resistant tuberculosis (MDR-TB) in Vietnam was 2.3% in 1996 [22]. Given that directly observed therapy short course (DOTS) coverage and case detection and cure rates in Vietnam have been exceeded WHO targets for many years, a fall in the incidence rate might have been expected. It is unclear why no such decline is evident, but lack of contact tracing, no treatment for latent TB infection, an unregulated private sector for TB treatment and an emerging HIV epidemic may all be possible contributors. The first HIV case was reported in Vietnam in 1990. Since then the number of reported HIV infections has escalated and the total number of people living with HIV was estimated to be 260,000 (range 150,000–430,000) at the end of 2005 [23]. While the national adult HIV prevalence was estimated to be 0.5% in 2005, it exceeds 1% in certain provinces.

In view of the high prevalence of tuberculosis and escalating HIV epidemic we conducted a prospective, observational cohort study to determine the clinical and microbiological features and outcome of HIV-infected patients presenting with TBM to the Hospital for Tropical Diseases, Ho Chi Minh City, Vietnam and to determine which baseline variables were associated with death.

## Methods

### Objectives

The aim of this study was to determine the clinical and microbiological features, outcome, and baseline variables predictive of death, in HIV-associated TBM.

### Setting and study participants

Patients were admitted the Hospital for Tropical Diseases, Ho Chi Minh City, Vietnam. This 500-bedded hospital acts as a primary, secondary and tertiary referral centre for infectious diseases, and serves the population of southern Vietnam (approximately 38 million).

### Inclusion and exclusion criteria

The study inclusion criteria were age ≥15 years, HIV infection, and clinically suspected TBM. Patients were excluded if there was microbiological evidence of another central nervous system (CNS) infection.

### Consent procedures

Patients who were fully conscious gave written informed consent. For patients who were unable to provide their own consent, written informed consent was obtained from their relative or, if no relative was available, from two independent physicians.

### Ethical approval

The study was approved by the Oxford Tropical Research Ethics Committee, UK, and the Scientific and Ethical Committee of the Hospital for Tropical Diseases, Ho Chi Minh City, Vietnam.

### Patient assessment

All patients underwent routine clinical and laboratory assessments, including CSF examination, chest radiography and cranial imaging, if possible.

Patients were defined as having definite or probable TBM according to the following criteria. Patients were classified as definite TBM if the CSF was smear positive for acid-fast bacilli and/or culture positive for *M. tuberculosis*. Patients were classified as probable TBM if they fulfilled study entry criteria and had at least one of the following four criteria: chest radiograph consistent with pulmonary tuberculosis; other specimens (e.g. sputum, lymph node, gastric washings) positive for acid-fast bacilli; evidence of extra-pulmonary tuberculosis; computed tomography (CT) or magnetic resonance imaging (MRI) evidence of tuberculous meningitis.

Severity of disease at presentation was assessed using the British Medical Research Council TBM grade [24]; grade I TBM is defined as a Glasgow coma score (GCS) of 15 with no focal neurology; grade II TBM as a GCS of 15 with a focal neurological deficit, or a GCS of 11 to 14 and grade III TBM is defined as a GCS of ≤10.

During the study period, patients were evaluated weekly for the first 3 months, then every 3 months until withdrawal, loss to follow-up, completion of TBM treatment, or death.

### Microbiological investigations

Up to 10mls of CSF was collected in a sterile Universal container and centrifuged at 4000xg for 15 minutes. Most of the supernatant was removed and the deposit was re-suspended in approximately 200 µl of supernatant. A portion of the deposit was examined by Gram, India ink and Ziehl-Neelsen stains. The remaining deposit was cultured on blood and chocolate agar for bacteria and fungi, and on Löwenstein-Jensen medium (Becton Dickinson) and in liquid 7H9 media (Mycobacterium Growth Indicator Tubes, Becton Dickinson) for mycobacteria. CSF cultures were incubated at 37°C for 12 weeks and examined weekly for growth. All positive CSF cultures were sent to the Tuberculosis Reference Laboratory at Pham Ngoc Thach Hospital, Ho Chi Minh City, for identification and drug susceptibility testing, using the 1% proportion method [25], [26].

### Drug treatments

Patients previously untreated for tuberculosis received isoniazid 5 mg/kg od po (maximum 300 mg/day), rifampicin 10 mg/kg od po (maximum 600 mg/day), pyrazinamide 25 mg/kg od po (maximum 2 g/day) and ethambutol 20 mg/kg od po (maximum 1.2 g/day) for 3 months followed by rifampicin, isoniazid and pyrazinamide at the same doses for a further 6 months. Patients who had previously been treated for tuberculosis also received intramuscular streptomycin 20 mg/kg (maximum 1.2 g/day) for the first three months, according to Vietnamese national guidelines. Patients received directly observed therapy during their inpatient stay (two to three months) and were followed up by the National Tuberculosis Programme after hospital discharge. Second-line antituberculous therapy was not available at the time of the study.

All patients received adjunctive dexamethasone therapy, unless there were contraindications to corticosteroids. The dose and duration of dexamethasone depended upon the TBM grade at presentation: patients with grade I TBM received 0.3 mg/kg/day, tapered over 6 weeks whereas patients with grade II or grade III TBM received 0.4 mg.kg/day tapered over 8 weeks [27].

All patients with baseline CD4 count <200 cells/mm^3^ were prescribed cotrimoxazole prophylaxis. Antiretroviral therapy was not routinely available at the time of the study. All patients who survived to hospital discharge were referred to the National HIV Programme for HIV treatment and care, according to local procedures.

### Data collection

Data were recorded onto individual case record forms, double entered in an Access database, and checked by the principal investigator, prior to statistical analysis.

### Statistical analysis

Clinical and microbial manifestations of HIV-associated TBM are described using medians with ranges or numbers with percentages. Median time to death was estimated using Kaplan-Meier methods and differences between TBM grade groups were compared using the log rank test. The Wilcoxon-Breslow-Gehan test of equality was used if the survival lines crossed. Univariate analysis was conducted using Cox regression to determine which presenting factors had a significant effect on time to death. To identify independent predictors for time to death, we entered all significant variables from the univariate analysis into a Cox regression model using a backward stepwise approach. The final model retained only those variables with a p-value less than 0.05. All statistical analyses were done using STATA, version 9 (StataCorp LP, College Station, Texas, USA).

### Role of the funding source

The corresponding author had full access to all the data and had final responsibility for the decision to submit for publication. The funding source had no role in study design, data collection and analysis, decision to publish, or preparation of the manuscript.

## Results

### Patient demographics and clinical features

58 patients were enrolled into the study between November 2004 and September 2005. Thirty nine (67.2%) patients died, eleven patients (19.0%) patients survived, two patients (3.4%) withdrew from the study and six patients (10.3%) were lost to follow-up.

The baseline demographic and clinical features are presented in the first column of table 1. The majority of patients were young, male, intravenous drug users. In terms of TBM disease severity at presentation, all three disease severity grades were represented although the majority of patients had grade III TBM. Most patients were aware of their HIV diagnosis and had other clinical features of HIV infection, including generalized lymphadenopathy, oral candidiasis and/or herpes zoster. All patients had World Health Organization HIV stage 4 disease by virtue of their TBM diagnosis. Only three patients were taking, or had previously taken, ART prior to study entry and four patients commenced ART therapy during the study period.

**Table 1:** Baseline demographic and clinical features of HIV TBM patients

Variable	All patients (n = 58)	Deaths (n = 39)	Survivors (n = 11)
Age in years, median (range)	26.5 (20–48)	25 (20–48)	32 (20–39)
Male	51 (87.9%)	34 (87.2%)	11 (100%)
Intravenous drug use	46/56 (82.1%)	31 (79.5%)	8 (72.7%)
Commercial sex worker	7/56 (12.5%)	4 (10.3%)	3 (27.2%)
Duration of symptoms, days, median (range)	11 (2–90)	14 (3–90)	7 (2–30)
Past history TB	12/55 (21.8%)	7/36 (19.4%)	4 (36.4%)
Known HIV positive	36/55 (65.5%)	25/37 (67.6%)	7 (63.6%)
Antiretroviral therapy	7 (12.1%)	1 (2.6%)	6 (54.5%)
Clinical features of HIV infection^1^	40 (69%)	26 (66.7%)	8 (72.7%)
Weight in kg, median (range)	49.5 (35–60)	48 (38–60)	50 (40–55)
Glasgow coma score	12 (3–15)	10 (3–15)	14 (8–15)
Focal neurology^2^	19 (32.8%)	16 (41%)	1 (9.1%)
TBM grade I	14 (24.1%)	7 (17.9%)	5 (45.5%)
TBM grade II	20 (34.5%)	10 (25.6%)	4 (36.4%)
TBM grade III	24 (41.4%)	22 (56.4%)	2 (18.2%)
Confirmed TBM	54 (93.1%)	37 (94.9%)	9 (81.8%)
Probable TBM	4 (6.9%)	2 (5.1%)	2 (18.2%)

Notes: Data are number (%) of patients, unless otherwise noted. Comparison of variables between deaths and survivors excludes patients who withdrew or were lost to follow-up. TBM grade = British Medical Research Council disease severity grade.

1 oral candidiasis, generalized lymphadenopathy, herpes zoster.

2 cranial nerve palsies, hemiplegia or paraplegia.

Comparison of the baseline demographic and clinical features between patients who died and those who survived are summarized in table 1. Data from patients who withdrew or were lost to follow-up were excluded from the comparison of deaths and survivors. Patients who died were more likely to have a prolonged duration of illness, lower Glasgow coma score, higher TBM grade and were less likely to be receiving antiretroviral therapy.

### Investigation results

The baseline laboratory variables are presented in the first column of table 2. Baseline laboratory abnormalities included low median values for haematocrit, blood lymphocyte percentage, serum sodium level and CD4 T-lymphocyte count. The median values for all other baseline blood tests were within the normal range.

**Table 2:** Baseline laboratory investigations in HIV TBM patients

Variable	Normal range	All patients (n = 58)	Deaths (n = 39)	Survivors (n = 11)
CD4 count, cells/mm^3^	500–1500	32 (2–285)^3^	31 (2–285)	37 (16–141)
Haematocrit %	37–47	34.8 (19.6–45.1)	34.9 (19.6–45.1)	35.4 (28.8–42.2)
Blood white cell count×10^9^/l	5–10	8.9 (2.7–21.9)	8.9 (2.7–20.6)	8.7 (3.7–21.9)
Blood neutrophil %	55–75	80.1 (41.6–92.9)	80.6 (41.6–92.9)	80.3 (61.2–91.2)
Blood lymphocyte %	20–40	10.0 (3–47.7)	9.9 (3–47.7)	8.8 (4.3–18.2)
Blood platelet count×10^9^/l	200–400	244 (75–604)	263 975–604)	235 (94–448)
Serum sodium mmol/l	35–145	127.8 (110–148)	125.7 (110–148)	135 (115–140)
Serum potassium mmol/l	3.5–5.0	3.9 (2.9–5.8)	3.9 (2.9–5.8)	3.9 (3.2–4.7)
Serum creatinine µmol/l	53–120	75.5 (9.4–276)	71 (9.4–276)	80 (53–164)
Serum bilirubin µmol/l	≤17	8 (1–47)^1^	8.3 (1–47)	8 (5–17)
Serum aspartate transaminase IU/l	≤37	41 (18–599)^2^	46 (18–599)	40 (22–158)
Serum alanine transaminase IU/l	≤40	42 (17–621)^2^	41.5 (17–621)	60 (22–240)

Notes: Data are expressed as median (range), unless otherwise noted. Comparison of variables between deaths and survivors excludes patients who withdrew or were lost to follow-up.

1 data from 52 patients.

2 data from 55 patients.

3 data from 50 patients.

Comparison of baseline laboratory variables between deaths and survivors are also shown in table 2. Data from patients who withdrew or were lost to follow-up were excluded from the comparison of deaths and survivors. Patients who died were more likely to have a low baseline median serum sodium level than those who survived.

The results of baseline CSF investigations are summarized in table 3. Abnormalities included a high CSF white cell count, high percentage of CSF neutrophils, elevated CSF protein level, low CSF to blood glucose ratio and elevated CSF lactate. Data from patients who withdrew or were lost to follow-up were excluded from the comparison of deaths and survivors. Patients who died were more likely to have a high CSF neutrophil percentage and a low CSF lymphocyte percentage.

**Table 3:** Cerebrospinal fluid and microbiology results in HIV TBM patients

Variable	Normal range	All patients (n = 58)	Deaths (n = 39)	Survivors (n = 11)
CSF WCC×10^9^ cells/l	≤5	438 (0–3960)	431 (0–3960)	340 (66–715)
CSF neutrophils %	0	63 (4–96)	70.5 (6–96)	30 (4–81)
CSF lymphocytes %	≤5	37 (4–96)	29.5 (4–94)	70 (18–96)
CSF protein g/l	≤0.4	1.3 (0.43–3.4)	1.23 (0.67–3.4)	1.41 (0.79–1.67)
CSF: blood glucose ratio	≥0.6	0.3 (0.1–0.8) 1	0.3 (0.1–0.6)	0.4 (0.3–0.8)
CSF lactate mmol/l	≤1.8	4.8 (1.7–10.2) 2	4.8 (1.7–10.2)	5.1 (2.7–6.7)
Positive Ziehl Neelsen stain		40 (69%)	32 (82.1%)	3 (27.3%)
Positive mycobacterial culture		51 (87.9%)	35 (89.7%)	9 (81.8%)
Isolate fully drug susceptible		21/46 (45.7%)	13/32 (40.6%)	3/8 (37.5%)
Isolate monoresistant		6/46 (13%)	4/32 (12.5%)	2/8 (25%)
Isolate polyresistant		15/46 (32.6%)	11/32 (34.4%)	3/8 (37.5%)
Isolate multidrug resistant		4/46 (8.7%)	4/32 (12.5%)	0/8 (0%)
Isolate resistant to streptomycin		24/46 (52.2%)	18/32 (56.3%)	5/8 (62.5%)
Isolate resistant to isoniazid		19/46 (41.3%)	15/32 (46.9%)	3/8 (37.5%)
Isolate resistant to rifampicin		4/46 (8.7%)	4/32 (12.5%)	0/8 (0%)

Notes: Data are expressed as median (range) or number (%). Comparison of variables between deaths and survivors excludes patients who withdrew or were lost to follow-up. CSF = cerebrospinal fluid. WCC = white cell count.

1 data from 57 patients.

2 data from 53 patients.

Forty (69%) CSF samples were smear positive for acid-fast bacilli by Ziehl-Neelsen stain and 51 (87.9%) CSF samples were culture positive for mycobacteria. Fourteen (24.1%) isolates were smear negative but culture positive; three (5.2%) isolates were smear positive and culture negative. Of the positive mycobacterial cultures, 49/51 (96.1%) isolates were identified as *M. tuberculosis* using the nitrate and reductase tests. Two isolates (3.9%) were smear and culture positive in our laboratory but were reported as ‘contaminated’ (mixed mycobacterial cultures) by the National Tuberculosis Reference Laboratory and were not further identified, according to local procedure.

Drug susceptibility testing results were available for 46 isolates (table 3). 21/46 (45.7%) CSF isolates were susceptible to all first-line drugs (rifampicin, isoniazid, pyrazinamide, ethambutol and streptomycin). 25/46 (54.3%) of CSF isolates had resistance to one or more first-line drugs. 6/46 (13%) isolates had mono-resistance (resistance to one first line drug), 14/46 had poly-resistance (resistance to two drugs other than rifampicin and isoniazid) and 4/46 (8.7%) isolates were MDR (resistance to at least rifampicin and isoniazid). In terms of resistance to individual drugs: 24/46 (52.2%) isolates were resistant to streptomycin; 19/46 (41.3%) isolates were resistant to isoniazid and 4/46 (8.7%) isolates were resistant to rifampicin, all of which were MDR.

Twelve patients had previously been treated for pulmonary or lymph node tuberculosis. Nine of these were CSF culture positive and eight had drug susceptibility testing performed. One isolate was MDR TB, three were resistant to isoniazid and streptomycin and one had streptomycin monoresistance. One patient who had a negative CSF culture on admission subsequently grew MDR-TB from pleural fluid. Comparison of CSF variables between deaths and survivors is summarized in table 3. The data from the patients who withdrew or were lost to follow-up were excluded from the comparison of deaths and survivors. CSF smear positivity, rifampicin resistance and multidrug resistance were more common in patients who died than in those who survived.

### Outcome

**Figure 1:**
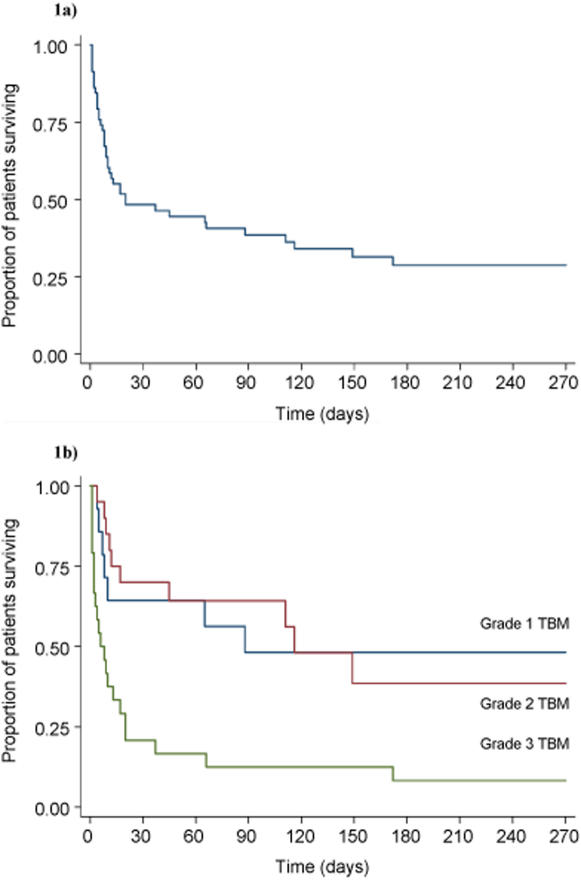
Kaplan-Meier survival estimate for HIV-associated TBM patients. a) survival curve for all patients. b) survival curve stratified according to TBM grade

Thirty nine (67.2%) patients died, with an overall median time to death of 20 days (range 1–172 days) [Figure 1a]. However, the median time to death varied significantly with TBM grade (p = 0.0002): 88 days for grade 1; 116 days for grade 2; and 6 days for grade 3 [Figure 1b]. While the survival functions for patient with TBM grade 1 and 2 were not significantly different (p = 0.93), patients with TBM grade 3 died significantly faster when compared with patient with TBM grade 1 (p = 0.006).

During the course of the study, 10 (17%) patients developed further AIDS-defining conditions e.g. *Pneumocystis jiroveci* pneumonia (n = 4), cryptococcal meningitis (n = 2), *Penicillium marneffei* bacteraemia (n = 2), oesophageal candidiasis (n = 1) and non-typhoidal salmonella bacteraemia (n = 1).

Univariate analysis of baseline characteristics identified seven factors that were associated with time to death: TBM grade [Hazard ratio (HR) 2.11, 95% confidence interval (CI )1.33 to 3.34], lack of antiretroviral therapy (HR 0.09, 95% CI 0.01 to 0.68), low baseline GCS (HR 0.85, 95% CI 0.78 to 0.93), low serum sodium (HR 0.93, 95% CI 0.89 to 0.97), high CSF neutrophil percentage (HR 1.02, 95% CI 1.01 to 1.04), low CSF lymphocyte percentage (HR 0.98, 95% CI 0.97 to 0.99) and CSF smear positive (HR 3.46, 95% CI 1.50 to 7.98).

The multivariate analysis identified three independent predictors for earlier time to death: increased TBM grade [adjusted hazard ratio (AHR) 1.73, 95% CI 1.08–2.76, p = 0.02], lower serum sodium (AHR 0.93, 95% CI 0.89 to 0.98, p = 0.002) and decreased CSF lymphocyte percentage (AHR 0.98, 95% CI 0.97 to 0.99, p = 0.003).

## Discussion

There is limited information on the influence of HIV infection on the clinical presentation, laboratory features and outcome of TBM. Most studies published to date have been small, retrospective and have compared the clinical features of TBM in HIV-infected and uninfected patients. Some studies have reported that HIV infection does not alter the presenting clinical features of TBM [28], [29], whereas others have suggested that HIV infection is associated with a higher rate of extra-pulmonary disease [30], extra-meningeal disease [31], [32] and radiological abnormalities on cranial imaging [32]

In terms of laboratory features of HIV-associated TBM, a previous study of showed that HIV infection was associated with a lower CSF leucocyte count and with isoniazid and streptomycin resistance [33] In another study, 21% of patients had an acellular CSF, 39.3% had CSF neutrophil predominance, and the CSF smear was positive in only 5.4% [31]. A prospective study of 123 Indian children (8/123 HIV infected) showed that HIV-infected children were more likely to have a haemoglobin <8 g/dl than uninfected children (OR 12, 95% CI 2.6–55.9, p = 0.001) [29].

There is also conflicting data on the effect of HIV infection on outcome in TBM. Some studies have failed to demonstrate a significant impact of HIV on inpatient mortality from TBM [2], [34]–[36], whereas others have reported significantly higher mortality rates in HIV-infected TBM patients [28], [37]. A South African study reported significantly more treatment failures in the HIV-infected group suggesting that HIV infection may influence the response to treatment and the likelihood of relapse [31].

The current study was designed as a prospective descriptive study, to define the clinical and microbiological features of patients with HIV-associated TBM in Vietnam. The patients were predominantly young, male, intravenous drug users, a finding consistent with previously reported epidemiological data from Vietnam [23]. Patients presented late with a prolonged duration of illness, severe TBM disease, as evidenced by a high proportion of patients with grade III TBM, and advanced HIV disease with low CD4 counts.

In contrast to previous studies [19], [31], [33], patients in the current study often had a high CSF cell count, with neutrophil predominance. Such findings are more commonly associated with acute bacterial meningitis and may mislead clinicians, resulting in a delayed or missed diagnosis of TBM. One possible explanation for this finding is that TBM may occur as a manifestation of primary infection in HIV-infected patients, as patients often have a miliary appearance on chest radiography. However, the CSF abnormality that was found to be associated with death was a low CSF lymphocyte percentage. One possible explanation is that patients with advanced HIV disease usually have low numbers of lymphocytes in the peripheral blood, which may be reflected in the low CSF lymphocyte count. In addition, TBM may stimulate increased HIV replication in the CNS compartment, resulting in destruction of CSF lymphocytes.

The other abnormality that was associated with death was a low serum sodium level, which may be a marker of severe TBM disease. The underlying cause of hyponatraemia in TBM is unknown but possible explanations include cerebral salt wasting syndrome, syndrome of inappropriate antidiuretic hormone secretion (SIADH), or hyponatraemic natriuretic syndrome [38].

The CSF smear and culture positivity rates were also higher than those reported from previous studies of HIV-associated TBM [19], [28], [31], [34], [35]. This may be because the CSF smears were performed, using large volumes of CSF (5–10 mls) by a dedicated technician in a research laboratory. Furthermore the quantity of bacilli seen in the CSF smear appeared to be higher, with a shorter time to detection of acid fast bacilli, in HIV-associated TBM than in HIV-negative TBM (data not shown). One explanation could be profound immune deficiency resulting in an attenuated immune response [39] and uncontrolled replication of *M. tuberculosis* within the subarachnoid space.

The majority of CSF isolates were identified as *M. tuberculosis*. Two isolates were reported as ‘contaminated’ by the regional reference laboratory, a recognized phenomenon in routine mycobacterial laboratories where there is high throughput of samples [40], [41].

The drug susceptibility testing results showed high rates of drug resistance with 54.3% resistance to one or more first line drugs and 8.7% multi-drug resistance. The rates of drug resistance in this study are considerably higher than those reported in the last Vietnamese National Drug Resistance Survey, although this was performed a decade ago. All patients with MDR TBM died, but streptomycin and/or isoniazid resistance were not associated with mortality, consistent with previously published data [42] Of the 25 cases of documented drug resistance only eight patients had previously been treated for tuberculosis, suggesting transmission of drug resistant TB in this population.

The outcome of HIV-associated TBM was dismal with a 67.2% mortality rate and a short time to death. The majority of patients presented with severe TBM and advanced HIV disease, both of which are known to be associated with poor prognosis [30]. At presentation all patients underwent investigations of the CSF for bacterial pathogens and cryptococcal meningitis, all which were negative. Investigations for viral CNS infections (e.g. polymerase chain reaction assays for human herpesviruses or JC virus) were not available at the time of the study. Thus, although the confirmed diagnostic rates for TBM were high in this study it was not possible to exclude all potential CNS pathogens. Cranial imaging was prohibitively expensive for most patients and was only performed at baseline in six patients (four CT scans and two MRI scan). The most common abnormalities were meningeal enhancement, hydrocephalus and cerebral infarcts. Neurosurgical intervention for TBM was not available at the time of the study.

Another factor that may have contributed to the high observed mortality rate was the delay in presentation to hospital. The median duration of symptoms prior to study entry was 11 days (range 2 to 90 days) and there was a non-significant difference between deaths and survivors. Once patients were admitted to the ward antituberculous therapy and adjunctive corticosteroids were commenced within 24 hours. The decision to commence TB treatment was based on a positive CSF smear result (69% of which were positive) or fulfillment of diagnostic criteria for probable TBM.

Unfortunately, it was not possible to determine the cause of death in all patients, particularly those who died after hospital discharge. In view of the observation that 17% of patients developed other AIDS defining illnesses during the study period, it is reasonable to speculate that some of these deaths may have been due to other undiagnosed opportunistic infections. Post-mortem examinations are rarely performed in Vietnam.

Patients presenting with HIV-associated TBM clearly warrant antiretroviral therapy and prophylaxis against opportunistic infections by virtue of their profound immunosuppression. Although antiretroviral therapy is becoming available in Vietnam access to treatment is difficult for patients admitted to hospital with their AIDS-defining illness. At present, access to treatment is restricted to ambulatory patients who are well enough to attend an outpatient clinic. Furthermore, there are significant delays between the diagnosis of HIV infection, referral to the clinic, adherence training and commencement of antiretroviral therapy. Thus in 2008, three years after the introduction of antiretroviral therapy funded by external donor agencies, only 20% of patients who warrant antiretroviral therapy at our hospital are actually receiving it.

In this study all patients who survived to hospital discharge were referred to the National HIV Programme for HIV treatment and care, according to local policy. The benefits of antiretroviral therapy are clearly demonstrated by the observation that six of the seven patients who received antiretroviral therapy survived. The one patient that died despite receiving antiretroviral therapy had MDR-TB isolated from pleural fluid.

In conclusion, HIV-associated TBM is a devastating disease and a number of formidable challenges remain. These include rapid diagnosis of drug-resistant tuberculosis, determining the optimal regimens and duration of treatment, determining the optimal time to initiate antiretroviral therapy, assessing the risks and benefits of adjunctive corticosteroid therapy in this already severely immunosuppressed population and perhaps, most importantly, ensuring universal free access to HIV treatment and care. Clinical trials to answer these questions are urgently required.
